# Genome-wide contributions of the MutSα- and MutSβ-dependent DNA mismatch repair pathways to the maintenance of genetic stability in *Saccharomyces cerevisiae*

**DOI:** 10.1016/j.jbc.2023.104705

**Published:** 2023-04-12

**Authors:** Lyudmila Y. Kadyrova, Piotr A. Mieczkowski, Farid A. Kadyrov

**Affiliations:** 1 Department of Biochemistry and Molecular Biology, Southern Illinois University School of Medicine, Carbondale, Illinois, USA; 2 Department of Genetics, Lineberger Comprehensive Cancer Center, University of North Carolina, Chapel Hill, North Carolina, USA

**Keywords:** DNA repair, DNA mismatch repair, genome integrity, DNA polymerase, MSH3, MSH6

## Abstract

The DNA mismatch repair (MMR) system is a major DNA repair system that suppresses both inherited and sporadic cancers in humans. In eukaryotes, the MutSα-dependent and MutSβ-dependent MMR pathways correct DNA polymerase errors. Here, we investigated these two pathways on a whole genome level in *Saccharomyces cerevisiae*. We found that inactivation of MutSα-dependent MMR increases the genome-wide mutation rate by ∼17-fold and loss of MutSβ-dependent MMR elevates the genome-wide mutation rate by ∼4-fold. We also found that MutSα-dependent MMR does not show a preference for protecting coding or noncoding DNA from mutations, whereas MutSβ-dependent MMR preferentially protects noncoding DNA from mutations. The most frequent mutations in the *msh6Δ* strain are C>T transitions, whereas 1- to 6-bp deletions are the most common genetic alterations in the *msh3Δ* strain. Strikingly, MutSα-dependent MMR is more important than MutSβ-dependent MMR for protection from 1-bp insertions, while MutSβ-dependent MMR has a more critical role in the defense against 1-bp deletions and 2- to 6-bp indels. We also determined that a mutational signature of yeast *MSH6* loss is similar to mutational signatures of human MMR deficiency. Furthermore, our analysis showed that compared to other 5′-NCN-3′ trinucleotides, 5′-GCA-3′ trinucleotides are at the highest risk of accumulating C>T transitions at the central position in the *msh6Δ* cells and that the presence of a G/A base at the −1 position is important for the efficient MutSα-dependent suppression of C>T transitions. Our results highlight key differences between the roles of the MutSα-dependent and MutSβ-dependent MMR pathways.

Mutations are necessary for evolution and to maintain the diversity of life. However, mutations also cause numerous diseases and can decrease fitness. Mutations arise in the genome in a nonrandom manner. The rate and distribution of mutations are affected by the chemical features of nucleotides, mutagens, DNA sequence contexts, nucleosome positions, chromatin states, transcription levels, replication timing, and origins of replication (1, 2, 3, 4, 5, 6, 7, 8, 9, 10, 11, 12). Living organisms have evolved elaborate molecular mechanisms to maintain mutation rates at levels that are compatible with life (4). The high-fidelity DNA synthesis at the replication fork and the DNA mismatch repair (MMR) system play significant roles in maintaining spontaneous mutation rates at low levels (13, 14, 15).

DNA polymerases α, δ, and ε that belong to the B family of DNA polymerases are responsible for replication of the bulk of nuclear DNA (16, 17). Another member of the B family of DNA polymerases, DNA polymerase ζ, also plays a role in DNA replication (18, 19). At the standard replication fork, DNA polymerases δ and α synthesize the lagging strand and DNA polymerase ε synthesizes the leading strand (16, 17, 20, 21). Nuclear DNA replication generates ∼1 to 2 errors for every 10^8^ nucleotides polymerized (10). The nucleotide selectivity and exonucleolytic proofreading by DNA polymerases are key contributors to the high-fidelity DNA synthesis (14, 15). The high-fidelity DNA polymerase selects correct nascent base pairs primarily based on geometry and its interactions with duplex DNA, with base-base hydrogen bonding providing a relatively small contribution to the selectivity (14). A significant fraction of DNA synthesis errors in eukaryotes is removed by the exonucleolytic activities of DNA polymerases δ and ε (17). The efficiency of exonucleolytic proofreading varies and depends on the sequence context (10, 22, 23, 24).

The MMR system corrects DNA polymerase errors in the form of base-base substitutions and 1 to 13 nt insertion/deletion loops that escape the proofreading activities of replicative DNA polymerases (25, 26, 27). Strand breaks in the daughter strands direct the MMR system to rectify replication errors (28, 29, 30, 31, 32). The efficiency of MMR is different at different genomic sites (3, 12). MMR increases the fidelity of DNA replication by ∼100 fold (10). Inactivation of the MMR system causes genetic instability and strongly predisposes humans and mice to the development of cancers (13, 33, 34, 35, 36, 37, 38, 39).

Eukaryotic MMR is initiated by the recognition of a mismatch by MutSα (MSH2-MSH6 heterodimer) (40, 41, 42) or MutSβ (MSH2-MSH3 heterodimer) (43, 44). MutSα is the primary mismatch recognition factor that recognizes both base-base mismatches and 1 to 13 nt insertion/deletion loops, and MutSβ is the second mismatch recognition factor that detects 1 to 13 nt insertion/deletion loops (25, 27, 40, 41, 42). After recognizing a mismatch, MutSα recruits MutLα in an ATP-dependent reaction (45, 46). The MutSα–MutLα complex interacts with replication factor C–loaded proliferating cell nuclear antigen to activate the endonuclease activity of MutLα (30, 47, 48). The activated MutLα endonuclease incises the discontinuous daughter strand in a reaction that is modulated by chromatin assembly factor-1–dependent histone (H3-H4)_2_ tetramer deposition (49, 50). A 5′ strand break produced by MutLα endonuclease is necessary for exonuclease 1 or a different enzyme to remove the mismatch (51, 52, 53, 54, 55, 56, 57).

Genetic studies of MMR have relied on the use of mutation reporters and whole genome sequencing (3, 7, 10, 23, 27, 33, 58, 59, 60, 61, 62). Although extensive genetic analyses of mutants that have been done at specific reporter loci have been critical for understanding the mechanisms of MMR, such analyses cannot often identify complex mutational signatures that could be observed in disease states. This limitation can be overcome by the use of whole genome sequencing–based approaches. Recent whole genome sequencing–based studies of MMR revealed that small insertion/deletions in yeast *mlh1* and *msh2Δ* cells are preferentially formed in noncoding DNA (7, 10, 62). Moreover, it was found that clustering of homopolymeric runs in a genome leads to the formation of mutation hot spots (7). While comprehensive genome-wide mutational analyses have been carried out for *Saccharomyces cerevisiae msh2Δ* strains (10, 62, 63), no such work was done for mutant strains that are defective in MutSα or MutSβ.

In this study, we examined the effects of MutSα-dependent and MutSβ-dependent MMR pathways on the maintenance of whole genome stability in *S. cerevisiae*. We found that both MMR pathways significantly suppress the genome-wide mutation rate. In addition, we determined that MutSβ-dependent MMR preferentially protects noncoding DNA from mutations and MutSα-dependent MMR does not have a bias for safeguarding noncoding or coding DNA against mutations. Compared to MutSβ, MutSα plays a more significant role in the suppression of 1-bp insertions, whereas MutSβ is more important than MutSα for the defense against 1-bp deletions and 2- to 6-bp indels. We also found that a single base substitution signature that we extracted from *S. cerevisiae msh6Δ* cells is similar to recently described single base substitution signatures of human MMR deficiency. Furthermore, our analysis shows that compared to the other genomic trinucleotides, 5′-GCA-3′ trinucleotides are at risk of acquiring C>T transitions at the middle position in the *msh6Δ* cells and that the presence of a purine base at the −1 position is necessary for the efficient MutSα-dependent repair of C>T transition-causing mismatches.

## Results

### Genome-wide spontaneous mutation spectra and mutation rates in *msh6Δ* and *msh3Δ* strains

**Figure 1:**
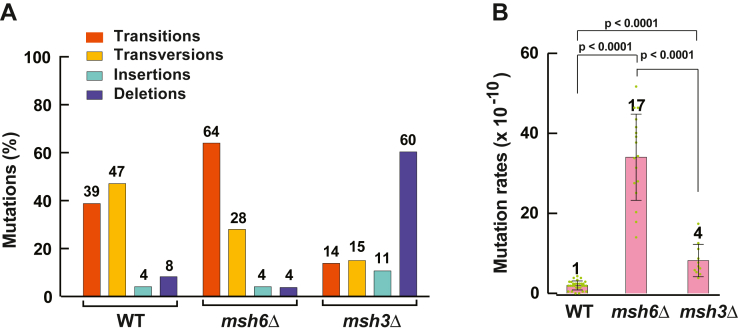
**Genome-wide mutation spectra and spontaneous mutation rates in *Saccharomyces cerevisiae msh6*Δ and *msh3*Δ strains.***A*, transitions, transversions, deletions, and insertions in the genome-wide mutation spectra of the WT, *msh6*Δ, and *msh3*Δ strains. *Numbers* above the bars are percentages. *B*, genome-wide spontaneous mutation rates in the WT, *msh6*Δ, and *msh3*Δ strains. The data are shown as the means ± SD (n_wt_ = 29, n_*msh6Δ*_ = 17, and n_*msh3Δ*_ = 11). Numbers above the bars are relative mutation rates. The mutation rates were calculated as described in Experimental procedures, and the *p* values were determined using the Mann–Whitney U two-tailed test (GraphPad Prism 6 software).

We began this study to better understand eukaryotic MMR on a genome-wide level. We initially performed mutation accumulation experiments in which we used 30 single-cell bottlenecks to passage multiple isolates of isogenic diploid *msh6Δ*, *msh3Δ*, and WT strains of the yeast *S. cerevisiae* on a solid-rich medium for ∼900 generations. We then identified mutations that accumulated in the nuclear genomes of the passaged isolates using whole genome DNA sequencing and generated the genome-wide mutation spectra. The mutation spectrum of *msh6Δ* strain consisted of transitions (64%), transversions (28%), insertions (4%), and deletions (4%) (Fig. 1*A*). The most common mutations in the spectrum of the *msh3Δ* strain were deletions (60%), and the remaining genetic alterations were insertions (11%), transitions (14%), and transversions (15%) (Fig. 1*A*). These data indicated that the genome-wide mutation spectrum of the *msh6Δ* strain significantly differs from the genome-wide mutation spectrum of the *msh3Δ* strain.

To clarify the contributions of the MutSα-dependent and MutSβ-dependent MMR pathways to the maintenance of genome stability in *S. cerevisiae*, we calculated genome-wide mutation rates in the WT, *msh6Δ*, and *msh3Δ* strains. Data analysis showed that the inactivation of MutSα-dependent MMR elevated the genome-wide mutation rate by 17-fold, and the loss of MutSβ-dependent MMR raised the genome-wide mutation rate by ∼4-fold (Fig. 1*B*). Thus, both MMR pathways significantly contribute to protecting the nuclear genome of *S. cerevisiae* from genetic changes.

### MutSα-dependent MMR does not preferentially protect coding or noncoding DNA from mutations

**Figure 2:**
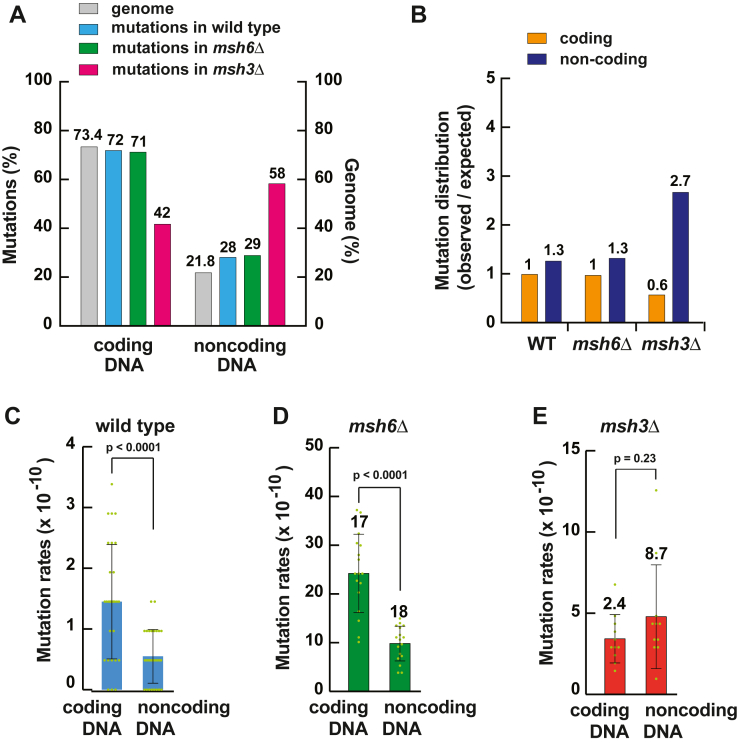
**Contributions of the MutSα-dependent and MutSβ-dependent MMR pathways to the defense of coding and noncoding DNA of *Saccharomyces cerevisiae* against mutations.***A*, mutations in the *msh3*Δ strain preferentially occur in noncoding DNA. *Numbers* above the bars are percentages. The *gray bars* indicate the proportions of the genome that are coding (73.4%) and noncoding (21.8%). The remaining 4.8% of the genome contain repetitive elements that cannot be uniquely mapped. *B*, bias toward the formation of mutations in noncoding DNA of the *msh3*Δ strain. The data are ratios of observed mutations to expected mutations. *Numbers* above the bars are ratios of observed mutations to expected mutations. *C*–*E*, mutation rates in coding and noncoding DNAs of the WT (*C*), *msh6*Δ (*D*), and *msh3*Δ (*E*) strains. The data are presented as the means ± SD (n_wt_ = 29, n_*msh6Δ*_ = 17, and n_*msh3Δ*_ = 11). Numbers above the bars are relative mutation rates. The mutation rates were calculated as described in Experimental procedures and did not take into account the proportion of the genome that is coding or noncoding. The Mann–Whitney U two-tailed test (GraphPad Prism 6 software) was utilized to calculate the *p* values. MMR, mismatch repair.

About 73% of nuclear DNA in *S. cerevisiae* is coding and the rest of the nuclear DNA is noncoding (64). We examined the distribution and rates of mutations in coding and noncoding DNAs of the *msh6Δ* strain. We determined that the distribution of mutations in the *msh6Δ* strain was very similar to the distribution of mutations in the WT strain (Fig. 2, *A* and *B*). We next calculated relative mutation rates in coding and noncoding DNAs of the *msh6Δ* strain. Relative mutation rates (*i.e.*, mutation rates that are relative to the corresponding WT mutation rates) in an MMR-deficient strain reflect MMR efficiencies. A higher relative mutation rate in an MMR-deficient strain is evidence of a higher efficiency MMR in the WT strain, and a lower relative mutation rate in the MMR-deficient strain is evidence of a lower efficiency MMR in the WT strain. We observed that in the *msh6Δ* strain, the relative mutation rates in coding DNA and noncoding DNAs were very similar (Fig. 2*D*). Collectively, these data indicated that MutSα-dependent MMR does not preferentially defend coding or noncoding DNA against mutations.

### MutSβ-dependent MMR preferentially protects noncoding DNA from mutations

We next analyzed the distribution and rates of mutations in coding and noncoding DNAs of the *msh3Δ* strain. We found that the distribution of mutations in the *msh3Δ* strain was different from the distribution of mutations in the WT strain (Fig. 2*A*). Specifically, the noncoding DNA of the *msh3Δ* strain contained 58% of mutations, whereas the noncoding DNA of the WT strain carried 28% of mutations. Additional analysis showed that the *msh3Δ* strain exhibited an ∼4-fold bias for the formation of mutations in noncoding relative to coding DNA (Fig. 2*B*). Unlike the *msh3Δ* strain, the WT strain displayed only a 20% bias for the formation of mutations in noncoding compared to coding DNA (Fig. 2*B*). In agreement with this, we established that in the *msh3Δ* strain, the relative mutation rate in noncoding DNA was 3.6 times higher than the relative mutation rate in coding DNA (Fig. 2*E*). Taken together, these findings revealed that MutSβ-dependent MMR preferentially prevents mutations in noncoding DNA.

### Effects of *msh3Δ* and *msh6Δ* on genome-wide rates of insertions and deletions

Both MutSα-dependent MMR and MutSβ-dependent MMR pathways rectify DNA polymerase errors in the form of small loops (25, 60). DNA loops on the parental strand, often referred to as deletion loops, give rise to deletions and DNA loops on the daughter strand, often called insertion loops, cause insertions. We analyzed the effects of *msh6Δ* and *msh3Δ* on genome-wide rates of deletions and insertions. We observed that in the *msh6Δ* strain, the rate of 1-bp deletions and the rate of 1-bp insertions did not significantly differ from each other and were ∼20 times higher than the rate of 2 to 6 bp indels (Table 1). We also observed that in the *msh3Δ* strain, the rates of 1-bp deletions and 2 to 6 bp indels were similar to each other and ∼11 to 14 times higher than the rate of 1-bp insertions (Table 1). In addition, our data indicated that deletion of *MSH6* does not significantly affect the rate of 2 to 6 bp indels and deletion of *MSH3* does not affect the rate of 1-bp insertions. Comparison of the genome-wide rates in the *msh6Δ* strain with those in the *msh3Δ* strain indicates that MutSα is more critical than MutSβ for the repair of 1-nt insertion loops and MutSβ plays a more significant role than MutSα in the removal of 1-nt deletion and 2 to 6 nt indel loops (Table 1).

**Table 1:** Rates of spontaneous deletions, insertions, and base substitutions in the WT, *msh6Δ*, and *msh3Δ* strains

Mutation type	Absolute mutation rate (×10^−11^)		
	WT	*msh6Δ*	*msh3Δ*
1-bp deletions	1.5^c^ (<0.17–4.8)	13^a,c^ (4.8–19)	31^a^ (19–44)
1-bp insertions	0.7^d,e^	14^b,d^ (4.8–19)	2.2^b,e^ (<0.44–4.8)
2–6 bp deletions and insertions	0.3^f,g^	0.57^f^	25^g^ (4.8–53)
Base substitutions	17^h,i^ (9.7–24)	313^i^ (227–401)	24^h^ (10–34)

The mutation rates that are marked with ^a,^^b,^^c,^^d,^ and ^g^ are statistically different from each other (^a^*p* = 0.0003, ^b^*p* < 0.0001, ^c^*p* < 0.0001, ^d^*p* < 0.0001, ^g^*p* < 0.0001, and ^i^*p* < 0.0001), whereas the mutation rates marked with ^e, f,^ and ^h^ are not statistically different from each other (^e^*p* > 0.99, ^f^*p* > 0.99, and ^h^*p* = 0.07). 95% confidence intervals are in parentheses.

### MutSα-dependent MMR is more efficient in preventing transitions than transversions

**Figure 3:**
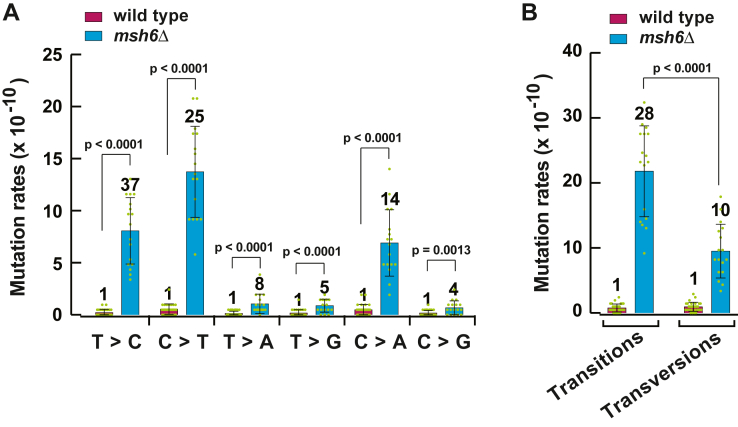
**Rates of different classes of base substitutions in WT and *msh6*Δ strains.** Rates of spontaneous C>T, T>C, T>A, T>G, C>A, and C>G mutations (*A*) and transitions and transversions (*B*) are shown. The data are shown as the means ± SD (n_wt_ = 29 and n_*msh6Δ*_ = 17). Numbers above the bars are relative mutation rates. The mutation rates were calculated as described in Experimental procedures, and the *p* values were computed using the Mann–Whitney U two-tailed test (GraphPad Prism 6 software).

Ninety-two percent of mutations in the spectrum of the *msh6Δ* strain are base substitutions (Fig. 1*A*). Previous analysis of relative genome-wide base substitution rates in an *msh2Δ* strain revealed evidence that *MSH2*-dependent repair corrects transition-causing mismatches more efficiently than transversions-causing mismatches (10). We analyzed the genome-wide rates of base substitutions in the *msh6Δ* strain. As shown in Figure 3*A*, the relative mutation rates were in the range of 14 to 37 for T>C and C>T transitions and C>A transversions and in the range of 4 to 8 for T>A, T>G, and C>G transversions. Overall, the relative rate of transitions was ∼3 times higher than that of transversions (Fig. 3*B*). These data provided evidence that compared to transversion-causing mismatches, transition-causing mismatches are removed by MutSα-dependent MMR more efficiently.

### Signatures of base substitution mutations in *msh6Δ* cells

**Figure 4:**
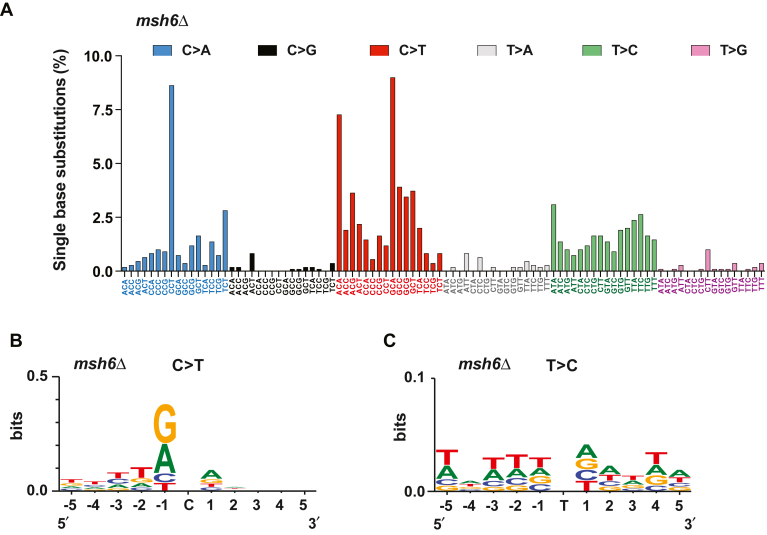
**Mutational signatures of yeast MutSα deficiency.***A*, a trinucleotide signature of base substitution mutations that were generated in *msh6*Δ cells. The mutational signature was obtained as described in Experimental procedures. *B* and *C*, signatures of C>T (*B*) and T>C (*C*) transitions that were produced in *msh6*Δ cells. The signatures were created in WebLogo (67) as detailed in Experimental procedures. Each of the aligned 11-nt sequences contains a mutated C (*B*) or T (*C*) base in the middle. To obtain the mutational signatures, 1102 (*A*), 482 (*B*), and 285 (*C*) sequences each containing a mutated nucleotide in the middle were aligned.

The discovery and analyses of mutational signatures have provided important insights into the nature of mutational processes in human cancers (4, 65, 66). We extracted a signature of base substitution mutations from the nuclear genomes of *msh6Δ* cells (Fig. 4*A*), which is based on the previously developed 96-substitution classification (65). The extracted signature has a prominent peak for C>A transversions at 5′-CCT-3′ sequences and two prominent peaks for C>T transitions at 5′-ACA-3′ and 5′-GCA-3′ sequences. The alterations within these three trinucleotides were responsible for ∼25% of all base substitutions in the *msh6Δ* cells. A significant fraction of base substitutions (∼20%) in the *msh6Δ* cells was comprised of transitions within 5′-ACG-3′, 5′-GCC-3′, 5′-GCG-3′, 5′-GCT-3′, 5′-ATA-3′, and 5′-TTC-3′ trinucleotides.

The most common base substitutions in the mutation spectrum of the *msh6Δ* cells were C>T transitions (Table S1). We utilized WebLogo (67) to further analyze sequence patterns in which C>T transitions were generated in the *msh6Δ* cells. The results indicated that in the MutSα-deficient cells, C>T transitions within the pentanucleotide sequence 5′-TTGCA-3′ were more frequent than within any other pentanucleotide sequence (Fig. 4*B*). We performed the same analysis to examine sequence contexts for T>C transitions that comprise the second most common class of substitutions in the mutation spectrum of the *msh6Δ* strains. As seen in Figure 4*C*, the most common pentanucleotide sequence in which T>C transitions were generated in the MutSα-deficient strains was 5′-TTTTA-3′.

### 5′-GCA-3′ trinucleotides are at an increased risk of accumulating C>T transitions in *msh6Δ* cells

**Figure 5:**
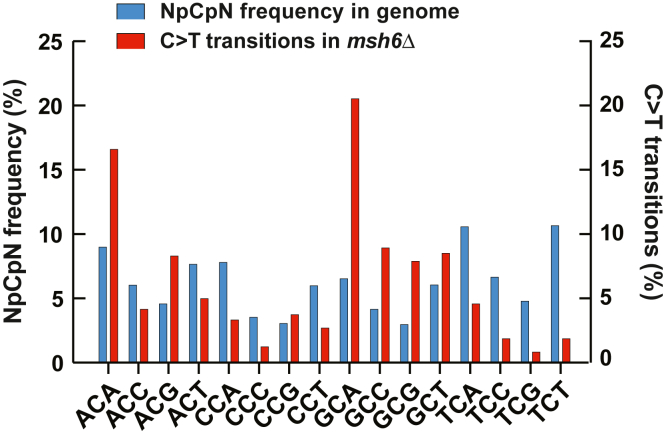
**The 5′-GCA-3′ sequences are at an increased risk of acquiring C>T transitions in the *msh6Δ* cells.** Comparison of the frequencies of different 5′-NCN-3′ trinucleotides in the yeast genome with the frequencies of C>T transitions (n = 482) in these trinucleotides in *msh6*Δ cells. The diploid genome of *Saccharomyces cerevisiae* that was used for calling mutations contains 8870546 5′-NCN-3′ trinucleotide sequences.

Twenty-one percent of C>T base substitutions in the mutation spectrum of the *msh6Δ* strains occurred within 5′-GCA-3′ trinucleotides (Fig. 5). We examined whether the increased frequency of C>T transitions was a result of increased frequency of 5′-GCA-3′ sequences in the yeast nuclear genome or a decreased fidelity of DNA synthesis of these trinucleotide sequences. The data showed that only 7% of 5′-NCN-3′ sequences in the yeast nuclear genome were 5′-GCA-3′ trinucleotides (Fig. 5). Further analysis indicated that compared to the other 15 kinds of 5′-NCN-3′ trinucleotide sequences, 5′-GCA-3′ sequences are at the highest risk of accumulating C>T transitions in the *msh6Δ* cells. Thus, this finding provided evidence that relative to the other 15 5′-NCN-3′ trinucleotides, 5′-GCA-3′ sequences are replicated by yeast DNA polymerases with the lowest fidelity.

### Sequence contexts for C>T mutations that MMR did not suppress

**Figure 6:**
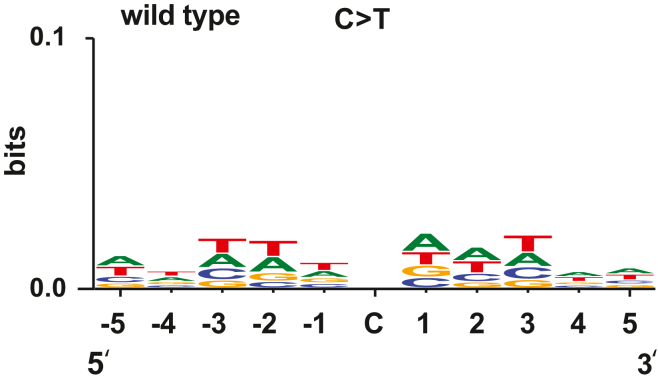
**A signature of C>T mutations that were generated in *Saccharomyces cerevisiae* WT cells.** The signature was generated as described in the legend for Figure 4, *B* and *C*. Three hundred sixty-two sequences each containing a mutated C base in the middle were aligned to create the mutational signature. The mutated sequences were identified in this and a previous study (68).

C>T transitions are the most common alterations in the mutation spectra of WT strains (68) (Table S1). Having found the consensus sequence for C>T transitions in *msh6Δ* cells (Fig. 4*B*), we analyzed sequence contexts for C>T transitions in the WT cells. The results showed that compared to other pentanucleotide sequences, C>T mutations in the WT cells most frequently occurred in the pentanucleotide sequence 5′-TTTCA-3′ (Fig. 6). Importantly, a majority of sequences in which C>T mutations were formed in the WT cells lacked a purine base at the −1 position (Fig. 6), whereas a majority of sequences in which C>T mutations were formed in the *msh6Δ* cells contained a G/A at the −1 position (Fig. 4*B*). This observation suggested that the presence of a G/A base at the −1 position is essential for efficient suppression of C>T mutations by MutSα-dependent MMR.

## Discussion

DNA polymerase errors are the primary source of mutations and have significant potential to alter metabolism and cause disease (4, 10, 34, 35, 69, 70). A major function of the MMR system in maintaining genetic stability is the correction of DNA polymerase errors (10, 13, 15, 71, 72). Previous elegant whole genome studies advanced our understanding of the factors that affect the generation of DNA polymerase errors and their removal by the MMR system (7, 10, 62, 63, 73). However, our understanding of the contributions of MutSα-dependent and MutSβ-dependent MMR pathways to the stability of genetic information on a genome-wide level is at an early stage. Here, we have shown that in *S. cerevisiae*, inactivation of MutSα-dependent MMR increases the genome-wide mutation rate by 17-fold and loss of MutSβ-dependent MMR elevates the genome-wide mutation rate by ∼4-fold (Fig. 1*B*). The observation that the genome-wide mutation rate in the *msh6Δ* strain is significantly higher than that in the *msh3Δ* strain is in line with the results of mutation reporter-based studies of the contributions of the two MMR pathways to the stability of coding DNA in *S. cerevisiae* (60, 74).

The *CAN1* forward mutation assay has been widely used to evaluate the effects of various mutated alleles on the spontaneous mutation rate in *S. cerevisiae*. The use of this mutation assay showed that the loss of *MSH6* in the haploid cells increases the spontaneous mutation rate in the coding *CAN1* DNA by 10- to 18-fold (60, 75). We have determined that the loss of *MSH6* in the diploid cells increases the mutation rate in coding DNA of the whole genome by 17-fold (Fig. 2*D*). Therefore, two different approaches, a mutation reporter-based approach and a next-generation sequencing–based approach, revealed similar contributions of the *MSH6* gene to maintaining the stability of coding DNA in *S. cerevisiae*.

Prior research demonstrated that *MSH2*-dependent MMR preferentially guards noncoding DNA from mutations (10, 62). In agreement with this, we have found that MutSβ-dependent MMR has a bias for protecting noncoding DNA from mutations (Fig. 2, *A*, *B* and *E*). Unlike MutSβ-dependent MMR, MutSα-dependent MMR does not show a bias for defending noncoding or coding DNA against mutations (Fig. 2, *A*, *B* and *D*). Notably, a bulk of mutations formed in *msh2Δ* strains are indels in noncoding DNA (10, 62). The loss of *MSH6* does not lead to a preferential formation of indels in noncoding DNA because in the absence of MutSα-dependent MMR, MutSβ-dependent MMR is sufficient to correct the majority of indel loops in noncoding DNA. The proofreading activities of the replicative DNA polymerases δ and ε are not efficient in the removal of frameshift intermediates that are formed during the replication of homopolymeric runs of ≥8 nts (22, 23). The inefficient correction of frameshift intermediates in the longer homopolymeric runs by replicative proofreading makes MMR the key process for maintaining the stability of the longer homopolymeric runs (23, 63). In agreement with this, we have observed that 76% of indels that are in homopolymeric runs of the *msh3Δ* strain and 86% of indels that are in homopolymeric runs of the *msh6Δ* strain are in the runs that are ≥8 nt long (Table S2). The importance of MMR for the stability of the longer homopolymeric runs implies that this versatile DNA repair pathway also corrects at least some other mismatches that are inefficiently removed by exonucleolytic proofreading. In accord with this idea, we have observed that of all 16 5′-NCN-3′ trinucleotides, 5′-GCA-3′ trinucleotides are at the highest risk of accumulating C>T transitions in the absence of MutSα-dependent MMR (Fig. 5) and that MutSα-dependent repair efficiently removes the C>T transition-causing mismatches that contain a purine base at the −1 position (Figs. 4*B* and 6).

Prior studies of MMR revealed that loss of *MSH3* leads to a preferential formation of 1-bp deletions in mutation reporters (27, 76). In line with this, we have determined that the *msh3Δ* strain accumulates 1-bp deletions at a genome-wide rate that is significantly higher than that of 1-bp insertions (Table 1). Furthermore, our data have shown that on a whole genome level, MutSβ is more critical than MutSα for the repair of 1-nt deletion loops, whereas MutSα plays a more significant role than MutSβ in the repair of 1-nt insertion loops (Table 1). It is unknown why the repair of 1-nt insertion loops is more dependent on MutSα, and the repair of 1-nt deletion loops shows a stronger dependency on MutSβ. Previous studies demonstrated that MutSβ-dependent repair of small deletion loops occurs *via* two subpathways, one of which involves MutLα endonuclease and the other depends on MutLγ endonuclease (77, 78, 79, 80). The existence of the two MutSβ-dependent subpathways for the repair of small deletion loops might explain why the repair of 1-nt deletion loops is more dependent on MutSβ.

It has been found that in many organisms including *S. cerevisiae* and primates, longer homopolymeric sequences are present in a far greater excess in noncoding relative to coding DNA (10, 63, 81, 82). Such an unequal distribution of longer homopolymeric runs among the two genomic categories is probably a result of purifying selection against frameshifts in coding DNA (82). It has also been found that in *S. cerevisiae*, (i) DNA polymerases more frequently generate 1-nt deletion loops in longer than shorter homopolymeric runs (23) and (ii) MutSβ-dependent MMR is more important than MutSα-dependent MMR for the protection from 1-bp deletions (Table 1). Taken together, these findings provide an explanation as to why MutSβ-dependent MMR in *S. cerevisiae* preferentially protects noncoding DNA from mutations. What is the biological significance of having an MMR pathway that is preferentially targeted to noncoding DNA? One possibility is that the cell wants to make sure that the stability of noncoding DNA is well maintained because noncoding DNA contains important regulatory elements such as promoters and is under less purifying selection pressure than coding DNA.

The MMR system removes biosynthetic errors that are produced by DNA polymerases α, δ, and ε (10, 74). However, the MMR system does not rectify errors of DNA polymerase ζ (75, 83). Approximately 80% of DNA polymerase ζ errors produce base-base mismatches (75), and this DNA polymerase does not contribute to the mutability of homopolymeric runs of ≥7 nts (Table S3). It might be that base-base mismatches generated by DNA polymerase ζ escape the MMR system because they are formed in DNA sequence contexts (18) that are not compatible with MMR and/or because they occur during late replication when replication factor C–loaded proliferating cell nuclear antigen (47), an essential MMR factor, is no longer present on the mismatch-containing DNA.

Our measurements of the rates of transitions and transversions in the *msh6Δ* strain have shown that the absolute genome-wide rates of transitions and transversions differ significantly from each other in the MutSα-lacking cells (Fig. 3*B*). We have also observed that the relative genome-wide rate of transitions is 28, while the relative genome-wide rate of transversions is 10 (Fig. 3*B*). These data indicate that the MutSα-dependent pathway rectifies ∼97% of transition-causing mismatches and ∼90% of transversion-causing mismatches. It remains unknown why the MutSα-dependent pathway more efficiently removes transition-causing than transversion-causing mismatches. It is possible that this difference exists because compared to transversion-causing mismatches, transition-causing mismatches are better recognized by MutSα and/or a significant fraction of the transversion-causing mismatches is generated by DNA polymerase ζ.

Several mutational signatures extracted from human cancers are associated with MMR deficiency (65, 66). One of these mutational signatures is SBS44 (66). Our analysis has shown that SBS44 and the yeast mutational signature of *MSH6* deficiency (Fig. 4*A*) share several similarities. First, both signatures mainly consist of C>T, T>C, and C>A substitutions. Second, in both mutational signatures, the most common C>T transition and the most common C>A transversion are within 5′-GCA-3′ and 5′-CCT-3′ trinucleotides, respectively. Third, 70% of C>T transitions in SBS44 (66) and 54% of C>T transitions in the mutational signature of yeast MMR deficiency are within 5′-ACA-3′, 5′-GCA-3′, 5′-GCC-3′, and 5′-GCT-3′ trinucleotides (Fig. 4*A*). Another cancer-derived mutational signature that is associated with MMR deficiency is SBS15 (66). It can be seen that ∼45% of C>T transitions in SBS15 and 38% of C>T transitions in the mutational signature of yeast MMR deficiency occurred within 5′-GCN-3′ trinucleotides (66) (Fig. 4*A*). Thus, there is a similarity between SBS15 and the mutational signature of yeast MMR deficiency. We have also noticed that the mutational signature of yeast MMR deficiency (Fig. 4*A*) is very similar to the trinucleotide mutational signatures that were extracted from human *msh2Δ*, *msh6Δ*, *and mlh1Δ* iPSC cells (84). The similarities between the mutational signatures provide evidence that base-base mismatches produced by human and budding yeast DNA polymerases in MMR-deficient cells generate similar patterns of substitution mutations.

A recent study that utilized targeted deep sequencing of *can1* sequences obtained a signature of base substitution mutations from yeast *msh6Δ* cells (76). The *can1*-based mutational signature takes into account the trinucleotide frequency in the *CAN1* gene and shares significant similarities with our mutational signature that was modified to reflect the trinucleotide frequency in the yeast genome (Fig. S1). In both signatures, the two largest contributors to the mutational signal are C>T transitions and C>A transversions. Furthermore, both signatures have a prominent peak that was formed by C>T transitions in 5′-GCG-3′. In addition to the similarities, there are some differences between the two mutational signatures (76) (Fig. S1). For example, the two strongest peaks that are present in our mutational signature (Fig. S1) are absent in the *can1*-based mutational signature; one of the peaks is a result of C>A transversions in 5′-CCT-3′ and the other is due to C>T transitions in 5′-GCA-3′.

In summary, we have found that the MutSα-dependent and MutSβ-dependent MMR pathways are required for genome-wide protection of genetic information from mutations in the yeast *S. cerevisiae*. We have also found that MutSα-dependent MMR does not have a bias for defending coding or noncoding DNA against mutations, while MutSβ-dependent MMR preferentially safeguards noncoding DNA against mutations. Furthermore, we have observed that a mutational signature that we have extracted from *S. cerevisiae msh6Δ* cells is similar to several recently described signatures of human MMR deficiency. In addition, our data have shown that the 5′-GCA-3′ trinucleotides are at risk of accumulating C>T transitions in the *msh6Δ* cells and that the presence of a purine base at the −1 position is important for the efficient prevention of C>T mutations by MutSα-dependent MMR.

## Experimental procedures

### *S. cerevisiae* strains and gene disruptions

Yeast WT strains that were used in this study are BY4741, BY4742, FKY1719, FKY1720, FKY1721, and E134 (*MAT*α *ade5-1 lys2::InsE-A*_*14*_ *trp1-289 his7-2 leu2-3112 ura3-52*) (23). The BY4741 and BY4742 strains are isogenic and have the S288C genetic background. FKY1719, FKY1720, and FKY1721 are diploid strains that were obtained by crossing of the haploid WT BY4741 (*MATa his3Δ1 leu2Δ0 met15Δ0 ura3Δ0*) and BY4742 (*MATα his3Δ1 leu2Δ0 lys2Δ0 ura3Δ0*) strains. The diploid *msh6Δ* (FKY2204, FKY2205, and FKY2206) and *msh3Δ* (FKY2219, FKY2220, and FKY2221) strains are isogenic to the diploid WT strains (FKY1719, FKY1720, and FKY1721). Gene replacements were generated by introducing PCR-amplified disruption cassettes (85) into yeast cells utilizing a lithium/PEG-based transformation procedure (86). The gene disruptions were confirmed by PCRs.

### Mutation accumulation, library preparation, and genome sequencing

Mutation accumulation experiments were carried out according to a previously developed method (10). Briefly, multiple isolates of the diploid WT, *msh3Δ*, and *msh6Δ* strains were subjected to 30 single-cell bottleneck passages (∼900 generations) on solid yeast peptone dextrose medium supplemented with 60 mg/l adenine and 63 mg/l uracil (YPDAU) at 30 °C. Samples of the yeast cultures that were at generations 0 and 900 were used to prepare glycerol stocks that were stored frozen at −80 °C.

Glycerol stocks of the yeast isolates that were at generations 0 and 900 were streaked as patches on solid YPDAU medium and incubated at 30 °C for 20 to 24 h. Yeast genomic DNAs were isolated from the patches using the MasterPure DNA purification kit (Lucigen). Whole genome DNA libraries were prepared utilizing the NEBNext Ultra II FS DNA Library prep kit (NEB) and NEBNext Multiplex Oligos for Illumina (NEB). The average size of genomic DNA inserts in the libraries was 500 bp. The libraries were analyzed and quantified using a TapeStation system (Agilent), and 150 bp paired-end sequencing was performed on NovaSeq 6000 and NextSeq 2000 sequencing systems.

### Analysis of mutation spectra and calculation of mutation rates

Paired-end reads obtained during the whole genome sequencing were processed with bcl2fastq Conversion Software (https://support.illumina.com/sequencing/sequencing_software/bcl2fastq-conversion-software.html), v 2.20.0 (Illumina) and aligned to *S. cerevisiae* S288C reference genome. Variants that had average base quality of ≥20, forward/reverse balance of ≥0.05, frequency of ≥35%, and sequencing coverage of ≥10× were called utilizing CLC Genomics Workbench (Qiagen). Variants that were present in the genome of a yeast isolate at generation 0 were excluded from the list of variants that were found in the genome of the same isolate at generation 900. We also excluded from the analysis variants that were within telomeres, long terminal repeats retrotransposons, and paralogous genes *FLO* which represent 4.8% of the yeast genome because variants within these repetitive elements cannot be uniquely mapped (10). Filtered variants were exported from CLC Genomics Workbench to Excel and pooled based on the genotype to generate the mutation spectra that were analyzed with the Excel Data Filter and Genome Browser tools (The *Saccharomyces* Genome Database).

Mutation rates (μ) per base pair per generation for any mutation type, *i*, in the genome or a genomic category were calculated using the following equation (10): *μ*_*bp,i*_ = *N*_*i*_*/gen/N*_*g*_, where *N*_*i*_ is the number of mutations of type *i*, *N*_*g*_ is the size the diploid *S. cerevisiae* genome (in bp) in which mutations were called, and *gen* is the total number of mutation accumulation generations for all isolates of the genotype. The size of *S. cerevisiae* diploid genome (*Ng*) in which mutations were called was 22,983,805 bp.

Spontaneous mutation rates at the *his7-2* and *lys2::InsE-A*_*14*_ loci of E134 and E134 *rev3*Δ strains were measured using fluctuation tests and Drake’s formula as previously described (9).

### Mutational signatures

The position of each of the analyzed mutations was mapped on the *S. cerevisiae* S288C reference genome sequence using CLC Genomics Workbench (Qiagen) and the Genome Browser tool (The *Saccharomyces* Genome Database). To generate the mutational signatures, DNA sequences each containing the mutated base in the middle were extracted from the reference genome. The trinucleotide sequences were sorted into the 96 different classes, each of which is based on the base substitution class and the two nucleotides surrounding the mutated nucleotide (65). The sorting of the extracted trinucleotide sequences was carried out with the Excel Data Filter tool. The sequence logos for C>T and T>C base substitutions were created using WebLogo 3 (https://weblogo.threeplusone.com/create.cgi) (67) as detailed below. For each sequence logo, 11-nt DNA sequences each containing the mutated base in the middle were entered into the sequence data input window of the WebLogo 3 web interface, and the graphical representations of nucleic acid multiple sequence alignments were created using the setting marked as CG composition of *S. cerevisiae* (38%) and the output format encapsulated postscript (vector).

## Data availability

Whole genome DNA sequencing data have been submitted to the National Center for Biotechnology Information Bioproject: Sequence Read Archive (PRJNA937627).

## Supporting information

This article contains supporting information.

## Conflict of interest

The authors declare that they have no conflicts of interest with the contents of this article.
