# Supplementary material for: Genome-wide contributions of the MutSα- and MutSβ-dependent DNA mismatch repair pathways to the maintenance of genetic stability in Saccharomyces cerevisiae
Source: J Biol Chem. 2023 Apr 12;299(5):104705. doi: 10.1016/j.jbc.2023.104705 (PMC10196800; doi:10.1016/j.jbc.2023.104705)
Supplement: Supporting Tables S1–S3 and Figure S1 [file mmc1.docx]

**Supporting Information**

**Table S1. Mutation counts in the wild-type, *msh6Δ*, and *msh3Δ* strains**

|  | **wild type** | ***msh6Δ*** | ***msh3Δ*** |
| --- | --- | --- | --- |
| **Isolates** | 29 | 17 | 11 |
| **Passages** | 870 | 510 | 330 |
| **Generations** | 26,100 | 15,300 | 9,900 |
| **Genome size** | 22,983,805 bp | | |
| **Mutation type** | **Number of mutations** | | |
| **Deletions of single A/T pairs** | 9 | 38 | 48 |
| **Deletions of single G/C pairs** | 0 | 6 | 23 |
| **Insertions of single A/T pairs** | 4 | 45 | 5 |
| **Insertions of single G/C pairs** | 0 | 4 | 0 |
| **2-6-bp deletions** | 1 | 2 | 42 |
| **2-6-bp insertions** | 1 | 0 | 15 |
| **T→C** | 13 | 285 | 7 |
| **C→T** | 33 | 482 | 19 |
| **T→A** | 8 | 37 | 8 |
| **T→G** | 10 | 31 | 5 |
| **C→A** | 29 | 243 | 13 |
| **C→G** | 10 | 24 | 2 |
| **other** | 2 | 1 | 0 |
| **Total** | 120 | 1,198 | 187 |

**Table S2. Distribution of indels in homopolymeric runs of the yeast *msh3Δ* and *msh6Δ* strains**

| Homopolymer run  length (bp) | Number of indels in the *msh3Δ* strain | Number of indels in the *msh6Δ* strain |
| --- | --- | --- |
| 2 | 0 | 0 |
| 3 | 1 | 0 |
| 4 | 3 | 2 |
| 5 | 2 | 2 |
| 6 | 9 | 0 |
| 7 | 4 | 3 |
| 8 | 6 | 5 |
| 9 | 5 | 5 |
| 10 | 8 | 3 |
| 11 | 6 | 12 |
| 12 | 9 | 12 |
| 13 | 2 | 12 |
| 14 | 4 | 8 |
| >15 | 19 | 19 |
| total | 78 | 83 |

**Table S3.** ***REV3* does not affect the stability of homopolymeric runs at *his7-2* and *lys2::InsE-A_14_***

| **Genotype** | **Mutation rate (x10^-8^)** | | | |
| --- | --- | --- | --- | --- |
|  | ***his7-2*** | | ***lys2::InsE-A_14_*** | |
|  | **Absolute rate** | **Relative rate** | **Absolute rate** | **Relative rate** |
| Wild type | 0.6  (0.5 – 1.2) | 1 | 17  (15 – 24) | 1 |
| *rev3*Δ | 0.6  (< 0.6 – 0.7) | 1 | 19  (14 – 27) | 1.1 |

The haploid *S. cerevisiae* strains were E134 (wild type) and its derivative. 95% confidence intervals are in parentheses.


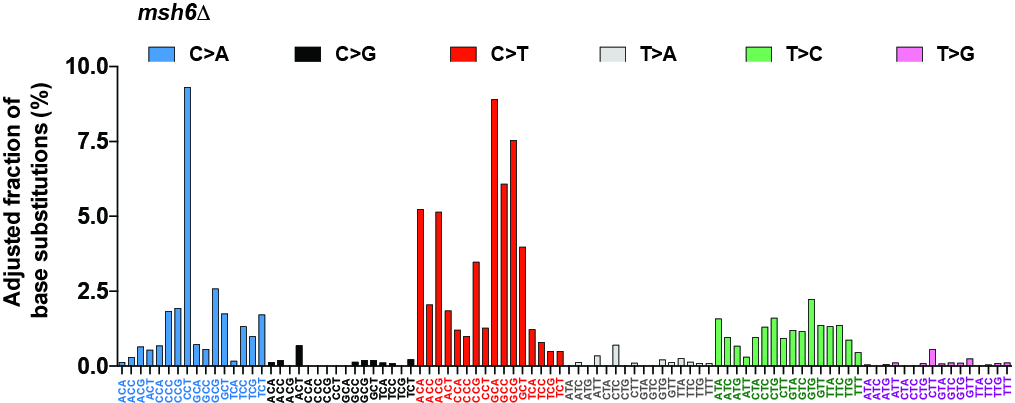


**Figure S1. A mutational signature that was obtained from the yeast *msh6Δ* cells**. The signature takes into account the frequencies of the different classes of trinucleotides in the S288C reference genome (The *Saccharomyces* Genome Database) lacking DNA sequences that cannot be uniquely mapped. To generate this mutational signature, the total number of base substitutions in each mutation class was divided by the number of the corresponding trinucleotides in the yeast genome. The data are presented as percentages.
